# Soil Microorganisms and Seaweed Application With Supplementary Irrigation Improved Physiological Traits and Yield of Two Dryland Wheat Cultivars

**DOI:** 10.3389/fpls.2022.855090

**Published:** 2022-06-01

**Authors:** Zahra Najafi Vafa, Yousef Sohrabi, Ghader Mirzaghaderi, Gholamreza Heidari

**Affiliations:** Department of Agronomy and Plant Breeding, Faculty of Agriculture, University of Kurdistan, Sanandaj, Iran

**Keywords:** drought stress, mycorrhiza, osmotic regulators, PGPR, wheat

## Abstract

To evaluate the effect of useful soil microorganisms and organic compounds on physiological characteristics and yield of two wheat cultivars under supplementary irrigation conditions, a study was conducted in the Agriculture Research Farm of Kurdistan University during the two cropping seasons of 2017–2018 and 2018–2019. A split–split plot-based study on a randomized complete block design with four replicates was used as an experimental design. The main factor was irrigation at three levels, including control without irrigation, supplementary irrigation in the booting stage, and supplementary irrigation in the booting and flowering stages. Two wheat cultivars, namely, Sardari and Sirvan, as sub-factors and application of bio-fertilizers in eight levels, including the use of bio-fertilizers containing: Mycorrhiza, Seaweed extract, Nitrozist and Phosphozist, Mycorrhiza + Nitrozist and Phosphozist, Seaweed extract + Nitrozist and Phosphozist, Mycorrhiza + Seaweed extract, Mycorrhiza + Nitrozist and Phosphozist + Seaweed extract, and non-application of bio-fertilizers, were considered as sub-factors. The results of both seasons of the experiment showed that the application of bio-fertilizers compared to the control treatment at all irrigation levels increased root volume, leaf relative water content (RWC), membrane stability index (MSI), and photosynthetic pigment content. The highest amount of H_2_O_2_, proline, and soluble carbohydrates were obtained in wheat under dry land conditions, and supplementary irrigation, especially two-time irrigation, significantly reduced the values of these traits. Supplementary irrigation also increased grain yield, so that in the conditions of two-time irrigation compared to the non-irrigation treatment (dry land), in the first and second seasons, the grain yield increased by 79.51 and 78.69%, respectively. Application of bio-fertilizers (Mycorrhiza + Nitrozist and Phosphozist + Seaweed extract) in comparison with the non-application of these fertilizers, due to increased root volume, RWC, MSI, and content of photosynthetic pigments, increased the grain yield in the first and second seasons of the experiment by 8.04 and 6.96%, respectively. As a result, suitable microorganisms and seaweed can improve wheat resistance mechanisms to water deficit, which along with using supplementary irrigation that saves water consumption improves plant growth and yield in areas faced with water shortage.

## Introduction

Wheat (*Triticum aestivum* L.) is one of the strategic crops in the world that plays a vital role in human nutrition and has always been considered an essential weapon in the fight against hunger (Sharma et al., 2019). Wheat is one of the crops with a large portion of the world’s agricultural fields and about 70% of total cereal production in Iran (Shariat Panahi et al., 2020). Crop production and providing the food needs of the world’s growing population have always faced challenges, and in the meantime, the impact of environmental stresses has been very significant. Abiotic stresses are the limiting factors for plant growth, yield, and geographical distribution. One of the most important abiotic stresses is drought stress, a serious obstacle to increasing agricultural production, and has become more important today due to climate change (Naderi et al., 2020). The grain filling stage in wheat under dry land conditions is often faced with decreasing rainfall and increased evaporation from the soil. The lack of water and increasing temperature during grain growth severely reduce the plants yield (Tricker et al., 2018). Therefore, finding solutions to improve plant growth conditions and reduce stress can be very important. Supplementary irrigation is a strategy that helps to improve plant growth and production during periods of low rainfall (Haghverdi et al., 2019). Supplementary irrigation means using a limited amount of water at the time of cessation of rainfall and in the sensitive plant growth conditions to provide sufficient water to continue plant growth and increase grain yield stability (Narimani et al., 2018). Among the physiological stages, flowering and grain filling are known as the most sensitive stages for irrigation. Supplemental irrigation in these stages can reduce stress and increase plant production (Gao et al., 2017). There is a strong relationship between soil moisture and the availability of plant nutrients. It has been seen that adequate soil moisture increases grain yield and quality (Kaplan et al., 2019). Optimal supply of plant needs leads to improved yield and increasing production of agricultural products, which is needed by the world’s growing population.

Use of chemical inputs, including chemical fertilizers, has been used for decades to improve the production and yield of crops. Nowadays, the excessive consumption of chemical fertilizers and environmental pollution caused by these fertilizers have severe adverse effects on the environment and necessitate using other sources, especially bio-fertilizers (Shariati et al., 2019). Bio-fertilizers are fertilizers containing a sufficient number of one or more beneficial soil organisms supplied with appropriate preservatives (Leylasi and Sarikhani, 2019). Using these microorganisms (which are prepared and marketed in the form of bio-fertilizers) in agricultural lands can increase their population in the root zone of plants. The plant benefits from the advantages of a sufficient population of these microorganisms in the soil. Bacteria and fungi are among the soil microorganisms affecting the growth and yield of plants.

Plant growth-promoting rhizobacteria (PGPR) are stable bio-fertilizers that assist and improve plant development by supplying minerals to the rhizosphere of stressed plants (Pagnani et al., 2018; Rehman et al., 2018; Hassan et al., 2019). Because bacteria, such as *Azotobacter* and *Azospirillum*, help in the absorption of water and nutrients. So, inoculating seeds with bacterium-based bio-fertilizers enhances plant yield significantly (Bilal et al., 2018). Studies suggest that the low yield of plants in chemical fertilizer treatments in dry land areas may be due to reduced plant access to nutrients, reduced soil moisture, or decreased nutrient uptake efficiency by plant roots (Asadi et al., 2019). Bio-fertilizers that contain growth-promoting rhizobacteria increase grain yield in dry land conditions by increasing plant access to important nutrients, such as nitrogen and phosphorus, and increasing root growth.

Arbuscular mycorrhiza fungi (AMF) are also beneficial soil microorganisms that significantly improve plants’ growth. Studies show that the symbiosis of AMF with plant roots increases the drought tolerance of plants (Liu et al., 2018; Jia-Dong et al., 2019). Substantial evidence in reducing the effects of drought stress by AMF exists in various crops, such as wheat, barley, corn, soybean, strawberry, and onion (Moradtalab et al., 2019). Inoculation of wheat plants with arbuscular mycorrhiza as a bio-fertilizer improves growth in infected areas and under stress conditions (Gupta et al., 2021). This fungus can help to increase the plant’s resistance to pathogens and its ability to compete with weeds (Mustafa et al., 2019; Khan et al., 2020). AMF is used as a bio-fertilizer. This fungus is a beneficial soil microorganism in the region of the plant rhizosphere that symbioses with the roots of 80% of plants (Jiang et al., 2017). In this symbiotic relationship, mycorrhiza consumes approximately 20% of the photosynthetic material released by plants for their growth and reproduction (Wipf et al., 2019). In contrast, it increases the plant’s ability to absorb nutrients, especially phosphorus and micronutrients, which helps to reduce phosphorus deficiency in the plant (Xiao et al., 2020). Extensive mycorrhizal hyphae can act as a highway for transferring water to plant cells (Zhang et al., 2019).

Nowadays, in addition to beneficial soil microorganisms, seaweed extract is also widely used as an organic fertilizer (Basavaraja et al., 2018). Researchers have demonstrated that seaweed extracts can increase the content of photosynthetic pigments in spinach and, as a result, improve the plant’s photosynthetic capacity and efficiency (Kulkarni et al., 2019). The researchers stated that the use of seaweed extract also has a favorable effect on the tolerance of plants to various environmental stress, and this substance plays an important role in protecting plants against stress (Elansary et al., 2017; Sharma et al., 2019).

The growing global population and the need to feed this growing population, on the one hand, and climatic changes, which in turn intensify the effects of environmental stress and further restricted the growth and yield of plants, have led to a focus on more research improving plant yield. Finding methods to lessen the impacts of stresses, such as drought, especially in arid and semi-arid regions of the world, and enhance the yield of strategic plants, such as wheat, is necessary, especially while considering the production, stability, and environmental effects. Therefore, this experiment aimed to investigate the effect of bio-fertilizers and supplementary irrigation on some physiological characteristics of two wheat cultivars to improve yield in dry land conditions.

## Materials and Methods

### Experimental Design

This research was carried out as a split–split plot based on a randomized complete block design with four replicates in the Agriculture Research Farm of Kurdistan University located in Dehgolan with coordinates of 47.18 degrees east and 35.18 degrees north with an altitude of 1,866 m above sea level (45 km east of Sanandaj city) during two cropping seasons of 2017–2018 and 2018–2019. Irrigation treatments were done at three levels: without irrigation (dry land), one-time supplementary irrigation at the flag leaf pod stage (booting), and two-time supplementary irrigation at the flag leaf pod and flowering stages, which were the main plots. Two wheat cultivars, namely, Sardari (dry land cultivar) and Sirvan (irrigation cultivar), were sub-factors. The seeds of the studied wheat cultivars were obtained from Sararud Dryland Agricultural Research Center.

Application of bio-fertilizers, including Mycorrhiza, Seaweed extract, Nitrozist and Phosphozist, Mycorrhiza + Nitrozist and Phosphozist, Seaweed extract + Nitrozist and Phosphozist, Mycorrhiza + Seaweed extract, Mycorrhiza + Nitrozist and Phosphozist + Seaweed extract, and non-application of bio-fertilizers, were considered as sub-factors.

### Analysis of Physicochemical Properties of Soil

Before sowing, seedbed preparation operations, including plowing, disk, leveling, and demarcation of the farm, were performed. A composite sample of field soil was prepared from the depth of 0–30 cm of soil and analyzed. The physical and chemical properties of the tested farm soil for two seasons of research are shown in Table 1.

**TABLE 1:** Physicochemical properties of the soil before sowing seed in two seasons of experiment (2017–2018 and 2018–2019).

Soil characteristics	2017–2018	2018–2019
Depth of soil (cm)	0–30	0–30
Soil texture	Laom	Clay
Electrical conductivity (ds m^–1^)	0.5050	0.4900
Ph	7.1700	7.2600
Total nitrogen (%)	0.1050	0.1370
Available K (mg Kg^–1^)	280	320
Available P (mg Kg^–1^)	8.6000	12.4000
Total organic carbon (%)	1.3700	0.7600
Available Fe (mg Kg^–1^)	9.8600	2.2000
Available Zn (mg Kg^–1^)	0.7900	0.8000

Soil texture was determined by hydrometer method (Gee and Bauder, 1986), and pH by saturated mud method using glass electrode (McLean, 1983), soil electrical conductivity (EC)^1^ in saturated extract using EC meter (Carter and Gregorich, 2006), organic carbon by Walk-Black method (Tandon, 1998), total nitrogen by Kjeldahl method (Bremner, 1965), absorbable potassium by ammonium acetate method and by flame gauge by Chapman and Pratt method (Chapman and Pratt, 1961), available phosphorus absorption by Olsen and Sommers (1982) method, and trace elements by Lindsay and Norvell (1978) method were measured. Also, weather data for two seasons of the experiment (2017–2018 and 2018–2019) are listed in Table 2.

**TABLE 2:** Monthly precipitation, average maximum and minimum relative humidity (Max RH_avg_ and Min RH_avg_), average maximum temperature (Max T_avg_), and average minimum temperature (Min T_avg_) for the growing season in 2017–2018 and 2018–2019.

	Year 2017–2018					Year 2018–2019				
Month	Precipitation (mm)	Max RH_avg_ (%)	Min RH_avg_ (%)	Max T_avg_ (°C)	Min T_avg_ (°C)	Precipitation (mm)	Max RH_avg_ (%)	Min RH_avg_ (%)	Max T_avg_ (°C)	Min T_avg_ (°C)
October	0.0200	44.4839	19.5806	21.1258	8.5419	13.6000	44.4839	19.5806	21.1258	8.67097
November	17.1700	71.2000	36	13.3433	3.4433	85.2000	71.2000	36	13.5767	3.5100
December	18.0300	69.6452	35.2581	10.7613	−0.8097	82.8000	69.6452	35.2581	7.71935	−3.4067
January	32.0600	80.4516	46	5.8161	−2.9871	67.4000	80.4516	46	6.1000	−3.1871
February	106.3300	81.1071	49.7143	8.3321	−0.7286	13.8000	81.1071	49.7143	8.7321	−0.1143
March	16.0200	64	27.7742	13.3694	1.4452	25.4000	79.9677	46.8387	9.9290	−0.9581
April	111	89.9000	36.4666	15.5167	3.7300	115.6000	81.9666	40.9000	12.4367	1.5667
May	70	98.6452	41.774	19.2548	6.4710	21.2000	84.8065	26.6774	22.1613	4.7129
June	0	82.4667	15.6833	28.6433	8.0500	0	73.30000	16.1000	30.3567	8.9667
Total	370.6300	–	–	–	–	425	–	–	–	–

### Plots Preparation

In this experiment, the dimensions of each sub-plot were 6.20 × 1.20 m^2^. Each experimental plot had six planting lines with a length of 6 m with a row spacing of 20 cm. The distance between replicates and the distance between the main plots was 2 m. The distance between the sub-plots was 60 cm. The distance between sub–sub plots was considered to be 40 cm. Wheat seeds were planted manually in autumn and at the first opportunity in early November. The time of harvest was early July to mid-July. The sowing density was 450 seeds/m^2^ and seeds were sown at a depth of 3 cm into the soil.

### Application of Arbuscular Mycorrhiza Fungi, Plant Growth-Promoting Rhizobacteria, and Seaweed Extract

Mycorrhiza fungus of *Glomus mosseae* species with 150 fungal spores per gram was prepared from a knowledge-based biotechnology center named Mycoperssica. According to the manufacturer’s recommendation, 40 g m^2^ was used as a strip next to the seeds at the time of sowing. Bacterial fertilizers Nitrozist with *Enterobacter cloacae* strain (having a role of atmospheric nitrogen stabilizer and plant growth stimulant similar to *Azospirillum* and *Azotobacter* bacteria) and Phosphozist with *Serratia marcescens* strain (*Phosphobacter*) in liquid form were used. Nitrozist and Phosphozist fertilizers have been prepared by knowledge-based (knowledge enterprise) Gostar Nozhan Company under the supervision of Bojnourd University Science and Technology Park. According to the manufacturer’s recommendation, 5 L was used for 300 kg of seeds by soaking the seeds. The seeds were inoculated with the mentioned biological fertilizers before sowing. The pretreatment time was 8 h. Foliar application of seaweed extract was performed in three growth stages: flag leaf pod stage (booting), spiking, and pollination stage. Fertilizer of seaweed extract (*Ascophyllum nodosum*) with the brand name Takamin Alga (made by Agri Techno Company from Spain) in liquid form (containing 16% algae extract, 7% organic matter, and 2.5% potassium) was used. The application of algae extract was 3 L per hectare (L/ha), and foliar application was done at sunset. Based on the soil analysis results, chemical fertilizers, including urea fertilizer at 50 kg/ha rate and phosphorus fertilizer at a rate of 20 kg/ha P_2_O_5_ from a triple superphosphate source, were applied completely before sowing the seeds. Weed control was done by manual weeding when needed.

### Irrigation of Experimental Plots

Irrigation of experimental plots was done by installing a volumetric water meter and drip irrigation using type pipes. To calculate the amount of irrigation water in the considered stages, after measuring the depth of root penetration from several plots, soil samples were prepared at the desired depth and their fresh weight was measured. Then, the samples were placed in an oven at 105°C for 24 h and their dry weight was calculated. Then, according to equation 1, the percent of weight moisture was determined, and then, according to equation 2, the volume of irrigation water was calculated (Alizadeh and Kamali, 2008).


(1) $$\mathrm{Percentage}\mathrm{by}\mathrm{weight}\mathrm{moisture}\left(\%\right)=\frac{\mathrm{Intial}\,\mathrm{soil}\,\mathrm{weight}-\mathrm{Soil}\,\mathrm{dry}\,\mathrm{weight}}{\begin{array}{c} \mathrm{initial}\,\mathrm{soil}\,\mathrm{weight} \end{array}}$$

(2) $$\mathrm{Irrigation}\,\mathrm{water}\,\mathrm{volume}=\frac{\left(\mathrm{Fc}-M\right)\times \rho \,\text{a}\times A\times \text{Ds}}{\text{Ea}}$$

In this regard,

V is the volume of irrigation in cubic meters,

Fc is the percentage of soil moisture in the field capacity,

M is the percentage of soil moisture before irrigation,

ρa is the specific gravity of the soil,

A is the area of the main plot,

Ds is the depth of root development in centimeters at the desired stage,

Ea is the irrigation efficiency.

Which in this experiment was considered 80–91% (Akhavan, 2015).

The volume of irrigation water for each stage of supplementary irrigation treatments was calculated according to the above formula.

### Measuring Traits

In this study, to measure traits, such as proline, soluble carbohydrates, hydrogen peroxide, chlorophyll a, b, total (a + b), and carotenoids, 2 weeks after pollination and at the same time as grain filling begins, taking into account the marginal effect in each of the plots, the flag leaves were randomly selected from five plants. The leaf specimens were wrapped in foil, immediately immersed in liquid nitrogen, and transported to a laboratory, whereupon they were refrigerated at a temperature of –40°C.

### Measurement of Root Volume

During the sampling stage, after pollination and coinciding with grain filling in all plots up to a depth of 30 cm from entirely uniform and homogeneous middle rows of wheat root samples (roots with soil and containing at least five plants per plot) were prepared. Samples prepared for washing were transferred to the laboratory in plastic bags. After complete soaking, the samples were gently rinsed with water pressure. A 500-ml graded cylinder was used to measure the root volume, and depending on the size of the root, 250 ml of water was poured into the graded cylinder. The root volume was measured in cm^3^ through the following equation (Naseri et al., 2018).


(3) Root⁢volume=Water⁢and⁢Root⁢Volume⁢(A)-Empty⁢Water⁢Volume⁢(B)

### Measurement of the Percentage of Root Colonization

A number of five plants from each plot were randomly selected during two growth stages before irrigation and 2 weeks after the last irrigation (end of root growth). After that, the roots were slowly and completely isolated from soils by washing. About 0.2 g of these was collected to prepare staining samples and determine the colonization percentage. These harvested microsamples were stained with a solution of ink and vinegar. Then, the stained roots were placed on a slide, and colonization was observed using a light microscope (Vierheilig et al., 1998). The amount of symbiosis in stained roots was determined with the help of the Norris et al. (1992) procedure.

### Measurement of Relative Leaf Water and Water Saturation Deficit

For each plot, about 4–5 pieces with an area of 2 cm from fresh leaf samples were separated, 2 weeks after pollination and coinciding with seed filling, and weighed quickly. The relative water content of leaf (RWC) and the amount of water saturation deficit (WSD) were calculated using equations 4 and 5 (Barr and Weatherley, 1962).


(4) $$\text{RWC}\left(\%\right)=\frac{\left(\mathrm{Fresh}\,\mathrm{leaf}\,\mathrm{weight}-\mathrm{Dry}\,\mathrm{weight}\,\mathrm{leaves}\right)}{\begin{array}{c} \left(\mathrm{Leaf}\,\mathrm{saturation}\,\mathrm{weight}-\mathrm{Dry}\,\mathrm{weight}\,\mathrm{leaves}\right) \end{array}}\times 100$$

(5) WSD(%)=100-RWC

### Measurement of Cell Membrane Stability Index

A total of two plants from each plot were randomly selected from the wet tissue of mature leaves 2 weeks after pollination (coinciding with seed filling) to measure the cell membrane stability index. Then, disks were removed from the leaves, and 0.1 g of the disks was washed with distilled water and placed in tubes that contain 10 ml of distilled water. The tubes were placed in a hot water bath at 40°C for 30 min and their electrical conductivity was cooled to 25°C after reading (C_1_). The falcon tubes were then kept in water at 100°C for 20 min and their electrical conductivity was read after cooling to 25°C (C_2_). Membrane stability index (MSI) was calculated as a percentage using equation (6) (Siddique et al., 2000).


(6) $$\text{MSI}\left(\%\right)=\left(1-\frac{\text{C}\,1}{\begin{array}{c} \text{C}\,2 \end{array}}\right)\times 100$$

C_1_ = Electrical conductivity after exposure to 40°C.

C_2_ = Electrical conductivity after exposure to 100°C.

### Measurement of Proline Content

To measure the proline content, the Bates et al. (1973)’s method was used. The concentration of soluble proline in toluene was determined using a spectrophotometer at 520 nm and according to the standard proline curve. To determine the proline concentration, a standard curve was drawn. After determining the amount of proline per gram of fresh leaf weight, the concentration of proline in mg g^–1^ dry weight was presented according to the ratio of dry weight to fresh leaf weight.

### Measurement of Soluble Carbohydrates

Antron method (Michalska et al., 2019) was used to measure leaf soluble carbohydrates. The frozen leaf samples prepared 2 weeks after pollination were used. The amount of absorption was measured at 630 nm with a spectrophotometer (UV- 2100SUV NEW JERSEY S2100). Finally, according to the water percentage of the samples, the data were expressed in mg g^–1^ dW.

### Measurement of Hydrogen Peroxide Content (H_2_O_2_)

To measure the amount of hydrogen peroxide, 0.3 g of leaves was milled in a porcelain pestle and mortar containing 5 ml of 0.1% trichloroacetic acid (TCA). The resulting extract was centrifuged at 10,000 rpm for 4 min at 4°C. Then, 250 μl of the supernatant from the centrifuge was mixed with 250 μl of phosphate buffer (pH = 7) mM and 500 μl of potassium iodide (KI) 1 M, and its absorbance was read at 390 nm with a spectrophotometer. The amount of hydrogen peroxide μmol per gram of fresh leaf tissue was determined using a standard curve (Alexieva et al., 2001).

### Measurement of Chlorophyll Content of a, b, Total (a + b), and Carotenoids

Lichtenthaler and Buschmann’s (2001) method was used to measure chlorophyll content. Finally, using equations 7, 8, 9, and 10, the content of chlorophylls a, b, total (a + b), and carotenoids was calculated and expressed in mg per gram of fresh leaf weight.


(7) $${\text{Chl}}_{\text{a}}\,\left(\text{ppm}\right)=\left(12.25\times \mathrm{A663}\right)-\left(2.79\times \mathrm{A646}\right)$$

(8) $${\text{Chl}}_{\text{b}}\,\left(\text{ppm}\right)=\left(21.21\times \mathrm{A646}\right)-\left(5.1\times \mathrm{A663}\right)$$

(9) $${\text{Ch}}_{\text{total}}\,\left(\text{ppm}\right)=\text{Chla}+{\text{Chl}}_{\text{b}}$$

(10) Carotenoid=((1000×A470)-(1.82×Chl)a-(85.02×Ch)b)/198.

### Grain Yield

To measure grain yield, after removing the marginal effects (removing 0.5 m from the beginning and end of planting lines and two side rows of plots), the plants in an area of 1 m^2^ were harvested for each experimental plot in the maturity stage. The seeds obtained from this area were weighed, and the grain yield was calculated in kilograms per hectare (kg ha^–1^) (Qayyum et al., 2021).

### Statistical Analysis of Data

Before analyzing the variance of the obtained data, the test of normality and uniformity of variance of the data error was checked by Minitab and SPSS software to perform appropriate data conversion if necessary. Then, ANOVA of data for each year was separately performed based on the relevant plan with the help of SAS statistical software (version 9.4). The uniformity test of variance of the obtained experimental errors for two seasons was performed using the Bartlett test for the studied traits. For traits whose variance of 2-year experimental errors was uniform, the combined data analysis of two seasons was performed. If the variance of the 2-year experimental errors was non-uniform, ANOVA was separately performed for each year. The means were compared by the least significant difference (LSD) method. The graphs were drawn using GraphPad software (version 9).

## Results

### Root Volume

**FIGURE 1:**
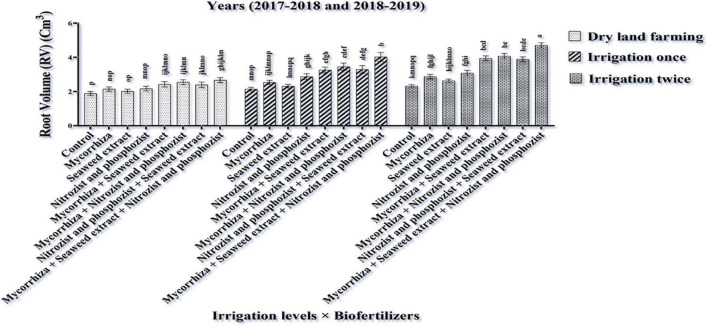
Mean comparisons of the dual interaction effect of irrigation × biofertilizers on root volume trait of wheat plant in two seasons of experiment (2017–2018 and 2018–2019). Means in each column followed by similar letter(s) are not significantly different at 1% probability level by the LSD test.

The combined ANOVA of root volume trait showed that the simple effects of irrigation and bio-fertilizer and the interaction effects of irrigation × bio-fertilizer and cultivar × bio-fertilizer were significant at a 1% level (Supplementary Table 1). The results indicate that the application of supplementary irrigation compared to dry land conditions caused a significant improvement in wheat root volume. Also, at all irrigation levels, the bio-fertilizer application significantly increased root volume compared to the control, and this increase was quite significant in the treatments containing a combination of bio-fertilizers (Figure 1).

**FIGURE 2:**
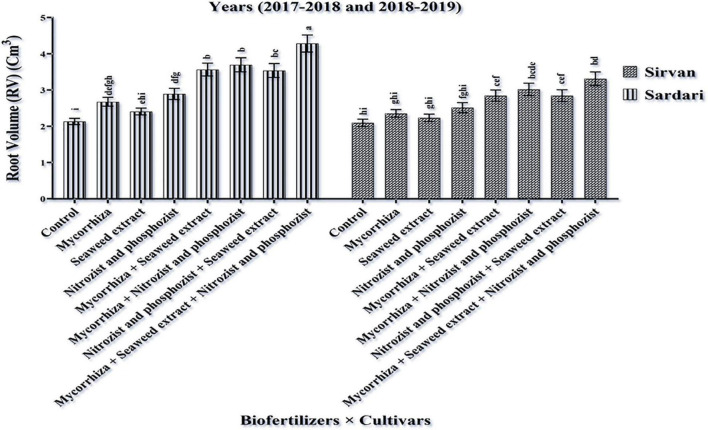
Mean comparisons of the dual interaction effect of biofertilizers × cultivars on root volume trait of wheat plant in two seasons of experiment (2017–2018 and 2018–2019). Means in each column followed by similar letter(s) are not significantly different at 1% probability level by the LSD test.

Based on the dual interactions between cultivars and bio-fertilizers (Figure 2), Sardari cultivar had the highest root volume (4.28 cm^3^) under Mycorrhiza + Nitrozist + Phosphozist + Seaweed extract fertilizers. The lowest root volume was allocated to the non-application of bio-fertilizers in the Sirvan cultivar (2.09 cm^3^) (Figure 2). This study explained that the root volume in the Sardari cultivar had a better response to bio-fertilizers.

### Determining the Percentage of Root Colonization

The analysis of variance of 2-year experimental data for the percentage of colonization showed that the main effects of irrigation, cultivar and bio-fertilizer were significant at 1% probability level (Supplementary Tables 2, 3). Also, in the second season of the experiment, the dual interaction of irrigation × bio-fertilizer on the mentioned trait was significant at 1% probability level (Supplementary Table 3). The results of comparing the mean in the first year of the experiment showed that two irrigations increased the percentage of root colonization compared to the dry land conditions by 54.90% (Table 3). A comparison of cultivars revealed that the Sirvan cultivar in the first and second seasons of the experiment (33.37 and 28.10%, respectively) than the Sardari cultivar (32.49 and 26.64%, respectively) had more root colonization (Tables 4, 5), which may be related to the greater dependence of this irrigated cultivar on the mycorrhizal fungus for its water supply. Also, the comparison of bio-fertilizer treatments displayed that plants treated with combined Mycorrhiza + Nitrozist and Phosphozist + Seaweed extract had the highest percentage of root colonization with an average of 36.04%. With an average of 29.52%, the lowest amount of this trait was obtained when no bio-fertilizer was applied (Table 6).

**TABLE 3:** Mean comparisons of simple main effects of irrigation levels on some traits in the wheat plant in 2017–2018.

Irrigation	PC (%)	H_2_O_2_ (μmol g^–1^FW)	ASC (mg g^–1^ dw)	WSC (mg g^–1^ dW)	Chlorophyll b (mg g^–1^ FW)	Yield Grain (kg ha^–1^)
Dry land farming	25.5503 ± 0.3369^c^	433.2900 ± 2.7866^a^	103.6500 ± 1.9533^a^	86.0511 ± 1.3772^a^	0.6557 ± 0.0179^c^	3481.8800 ± 72.6356^c^
One-time irrigation	33.6546 ± 0.2855^b^	275.9300 ± 5.9063^b^	68.1914 ± 1.1885^b^	68.4265 ± 1.7151^b^	1.0182 ± 0.0229^b^	5321.2800 ± 88.5954^b^
Two-time irrigation	39.5782 ± 0.2866^a^	158.8600 ± 3.3983^c^	42.1241 ± 0.8753^c^	30.3260 ± 1.4998^c^	1.2535 ± 0.0297^a^	6250.3800 ± 65.7888^a^

*The data are means of four replicates ± SE. Means in each column followed by similar letter(s) are not significantly different at 1% probability level by the LSD test. PC, percentage of colonization; ASC, alcohol-soluble carbohydrate; WSC, water-soluble carbohydrates.*

**TABLE 4:** Mean comparisons of simple main effects of cultivar on some traits in the wheat plant in 2017–2018.

Cultivars	PC (%)	RWC (%)	WSD (%)	H_2_O_2_ (μmol g^–1^ FW)	ASC (mg g^–1^ dw))	WSC (mg g^–1^ dW)	MSI (%)	Chlorophyll b (mg g^–1^ FW)	Carotenoid (mg g^–1^ FW)	Yield Grain (kg ha^–1^)
Sardari	32.4882 ± 0.6391^b^	80.2349 ± 0.8443*^a^*	19.7651 ± 0.8440^b^	267.9200 ± 12.2558^b^	75.0127 ± 2.8058*^a^*	67.0460 ± 2.4498*^a^*	72.4291 ± 0.9606*^a^*	0.9050 ± 0.0294^b^	3.6182 ± 0.1085^b^	4558.3400 ± 123.0040^b^
Sirvan	33.3672 ± 0.6377*^a^*	78.1694 ± 0.8649^b^	21.8306 ± 0.8649*^a^*	310.8000 ± 11.3591*^a^*	67.6302 ± 2.8035^b^	56.1564 ± 2.8111^b^	61.8194 ± 1.0119^b^	1.0467 ± 0.0327*^a^*	4.0249 ± 0.1113*^a^*	5477.3400 ± 126.9110*^a^*

*The data are means of four replicates ± SE. Means in each column followed by similar letter(s) are not significantly different at a 1% probability level by the LSD test. PC, percentage of colonization; RWC, relative water content; WSD, water saturate deficit; ASC, alcohol-soluble carbohydrate; WSC, water-soluble carbohydrates, cell membrane stability index.*

**TABLE 5:** Comparison of the mean of the dual interaction of cultivar and irrigation on some studied traits of wheat in 2017–2018.

Cultivars	PC (%)	RWC (%)	WSD (%)	H_2_O_2_ (μmol g^–1^ FW)	WSC (mg g^–1^ dW)	MSI (%)	Chlorophyll a (mg g^–1^ FW)	Total chlorophyll (a + b) (mg g^–1^ FW)	Carotenoid (mg g^–1^ FW)	Yield grain (kg ha^–1^)
Sardari	26.6352 ± 0.6238^b^	75.6373 ± 1.0048^a^	24.3627 ± 1.0048^b^	335.0600 ± 13.7432^b^	72.81530 ± 2.2915^a^	67.4005 ± 1.0470^a^	1.6804 ± 0.0665^b^	2.4028 ± 0.0842^b^	2.9687 ± 0.1020^b^	4437.9600 ± 115.7710^b^
Sirvan	28.1043 ± 0.6355^a^	73.0565 ± 0.9094^b^	26.9435 ± 0.9094^a^	385.6700 ± 13.9781^a^	66.9949 ± 2.2418^b^	56.9254 ± 1.1032^b^	1.8851 ± 0.0520^a^	2.8126 ± 0.0842^a^	3.5086 ± 0.0978^a^	5055.0800 ± 119.9590^a^

*The data are means of four replicates ± SE. Means in each column followed by similar letter(s) are not significantly different at a 1% probability level by the LSD test. PC, percentage of colonization; RWC, relative water content; WSD, water saturate deficit; WSC, water-soluble carbohydrates, MSI, cell membrane stability index.*

**TABLE 6:** Mean comparisons of simple main effects of bio-fertilizers on some traits in the wheat plant in 2017–2018.

Bio-fertilizers	PC (%)	H_2_O_2_ (μmol g^–1^FW)	ASC (mg g^–1^ dw)	WSC (mg g^–1^ dW)	Chlorophyll b (mg g^–1^ FW)	Yield grain (kg ha^–1^)
Control	29.5150 ± 1.2869*^f^*	299.5000 ± 24.3105^a^	63.1738 ± 5.0533^d^	53.3031 ± 5.0846^d^	0.8577 ± 0.0554^d^	4837.0800 ± 270.9100^c^
Mycorrhiza	32.9262 ± 1.15115^c^	294.8800 ± 24.5182^a^	67.7647 ± 5.45874^cd^	58.8620 ± 5.3618^c^	0.9703 ± 0.0658^abcd^	4933.3300 ± 269.4450^bc^
Seaweed extract	30.7321 ± 1.3146^e^	294.3900 ± 24.6876^ab^	70.6709 ± 5.8980^bc^	59.5469 ± 5.5392^c^	0.9364 ± 0.0603^bcd^	4941.2500 ± 274.3560^bc^
Nitrozist and Phosphozist	31.8008 ± 1.2797^d^	295.3300 ± 24.6760^a^	70.9710 ± 5.4919^bc^	61.0761 ± 5.4760^bc^	0.9146 ± 0.0584^cd^	4977.0800 ± 273.6690^bc^
Mycorrhiza + Seaweed extract	34.5146 ± 1.15659^b^	288.0700 ± 24.7057^bc^	71.1124 ± 5.1776^bc^	61.8948 ± 4.8866^bc^	1.0131 ± 0.0675^abc^	5073.3300 ± 266.8200^ab^
Mycorrhiza + Nitrozist and Phosphozist	34.8011 ± 1.1498^b^	283.0400 ± 24.0761^cd^	72.3477 ± 5.7602^bc^	63.6049 ± 5.3685^bc^	1.0448 ± 0.0677^ab^	5042.3200 ± 273.6690^ab^
Nitrozist and Phosphozist + Seaweed extract	33.0967 ± 1.2287^c^	282.9800 ± 24.2979^cd^	75.8362 ± 6.4966^ab^	64.8263 ± 5.8335^ab^	1.0003 ± 0.0655^abc^	5112.0800 ± 266.8200^ab^
Mycorrhiza + Seaweed extract + Nitrozist and Phosphozist	36.0350 ± 1.2451^a^	276.6900 ± 24.2613^d^	78.6946 ± 5.8842^a^	69.6955 ± 5.4267^a^	1.0694 ± 0.0645^a^	5226.2500 ± 262.6360^a^

*The data are means of four replicates ± SE. Means in each column followed by similar letter(s) are not significantly different at 1% probability level by the LSD test. PC, percentage of colonization; ASC, alcohol-soluble carbohydrate, WSC, water-soluble carbohydrates.*

**FIGURE 3:**
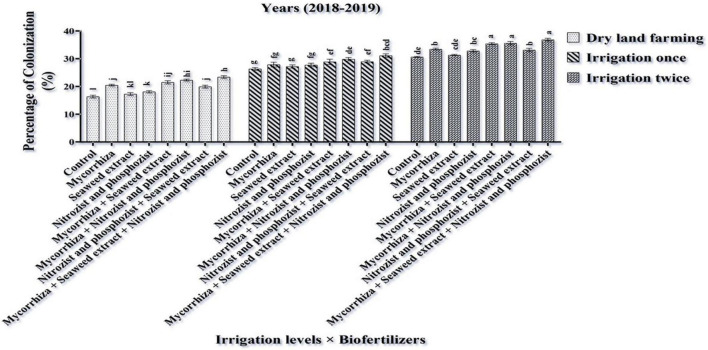
Mean comparisons of the dual interaction effect of irrigation × biofertilizers on colonization percentage trait of wheat plant root in two seasons of experiment (2017–2018 and 2018–2019). Means in each column followed by similar letter(s) are not significantly different at 1% probability level by the LSD test.

Comparing the mean of dual irrigation × bio-fertilizer interaction, it was found that supplementary irrigation increased the percentage of wheat root colonization. At all irrigation levels, the application of bio-fertilizers improved the amount of this trait. A combination of two-time supplementary irrigation and the use of bio-fertilizers (Mycorrhiza + Nitrozist and Phosphozist + Seaweed extract) achieved the highest level of root colonization (36.87%) compared to the controls (16.37%) (Figure 3). In this study, the combination of bio-fertilizers (Mycorrhiza + Nitrozist and Phosphozist + Seaweed extract) successfully colonized the roots. It helped to improve the growth of wheat cultivars (Sardari and Sirvan) under drought stress conditions.

### Relative Leaf Water Content and Water Saturation Deficit

The main effects of irrigation, cultivar, and bio-fertilizer at 1% probability level and dual irrigation interactions × bio-fertilizer were significant in the first year at 1% probability level and the second year at 5% probability level on the mentioned traits, according to the results of the ANOVA of the first- and second-year data of the experiment for RWC and WSD (Supplementary Tables 2, 3). The results of this study revealed that RWC in the first year and the second year of the experiment was higher in the Sardari cultivar (80.23 and 75.64%, respectively) than in the Sirvan cultivar (78.17 and 73.06, respectively) (Tables 4, 5).

**FIGURE 4:**
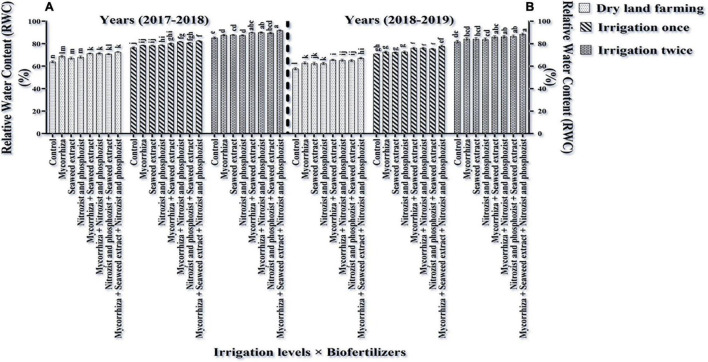
Mean comparisons of the dual interaction effect of irrigation × biofertilizers on RWC trait in wheat plant in two seasons of experiment **(A,B)** (2017–2018 and 2018–2019). Means in each column followed by similar letter(s) are not significantly different at 1% probability level by the LSD test.

**FIGURE 5:**
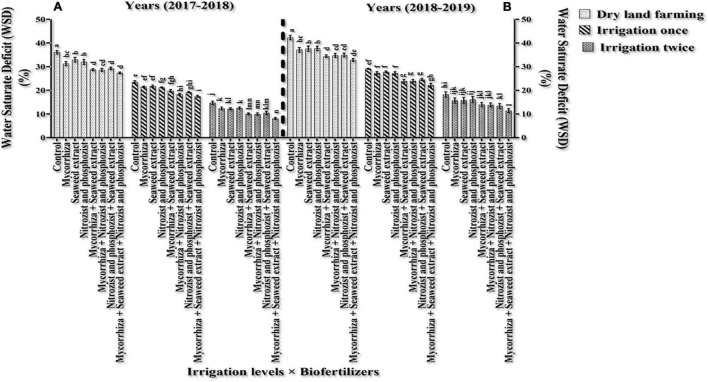
Mean comparisons of dual interaction effect of irrigation × biofertilizers on WSD trait in wheat plant in two seasons of experiment **(A,B)** (2017–2018 and 2018–2019). Means in each column followed by similar letter(s) are not significantly different at 1% probability level by the LSD test.

The results of comparing an average of interaction irrigation × bio-fertilizers (Figures 4, 5) illustrate that supplementary irrigation and increasing the number of irrigations with the bio-fertilizer application, especially in the simultaneous use of several bio-fertilizers, improved RWC and reduced WSD in wheat. The highest RWC content, with an average of 91.89 and 88.59%, respectively, was related to bio-fertilizers (Mycorrhiza + Nitrozist and Phosphozist + Seaweed extract) under two-time irrigation conditions in the first year and the second year of the experiment, respectively. The lowest values, with an average of 63.93 and 57.73%, respectively, were obtained in the control treatment (non-application of bio-fertilizers and irrigation) (Figure 4). The lowest amount of WSD in the first and second seasons of the experiment was obtained in two-time irrigation and bio-fertilizers (Mycorrhiza + Nitrozist and Phosphozist + Seaweed extract) conditions, with an average of 8.11 and 11.41%, respectively. The highest amounts were related to the condition of dry land and the non-application of bio-fertilizers, with an average of 36.07 and 42.27%, respectively.

A comparison of the mean of experimental data showed that the Sardari cultivar had the lower rate of WSD during the first and second seasons (with an average of 19.77 and 24.26%, respectively) in comparison with the Sirvan cultivar (with an average of 21.83 and 26.94%, respectively) (Tables 4, 5), which indicates the water situation in the Sardari cultivar is better than the Sirvan cultivar.

### Hydrogen Peroxide (H_2_O_2_)

The ANOVA of the first- and second-year data showed that the simple effects of irrigation treatments, cultivar, and bio-fertilizer had a significant effect at a 1% probability level on the H_2_O_2_ trait (Supplementary Tables 2, 3). In the second year of the experiment, the dual interaction of irrigation × bio-fertilizer was significant at a 1% probability level for this trait (Supplementary Table 4). Results of the first year of the experiment displayed that the highest amounts of hydrogen peroxide were obtained in dry land conditions (with an average of 433.29 μmol g^–1^ FW) and supplementary irrigation, especially two-time irrigation (with an average of 158.86 μmol g^–1^ FW) caused a significant reduction in the values of this trait (Table 3). The results of the first and second seasons (Tables 4, 5) showed that the highest amount of hydrogen peroxide in the Sirvan cultivar (310.80 and 385.67 μmol g^–1^ FW, respectively) and the lowest amount of hydrogen peroxide in the Sardari cultivar (267/92 and 366.06 μmol g^–1^ FW) were observed. So that in the Sirvan cultivar, the amount of hydrogen peroxide was about 16 and 15%, respectively, which was higher than in the Sardari cultivar (Tables 4, 5).

**FIGURE 6:**
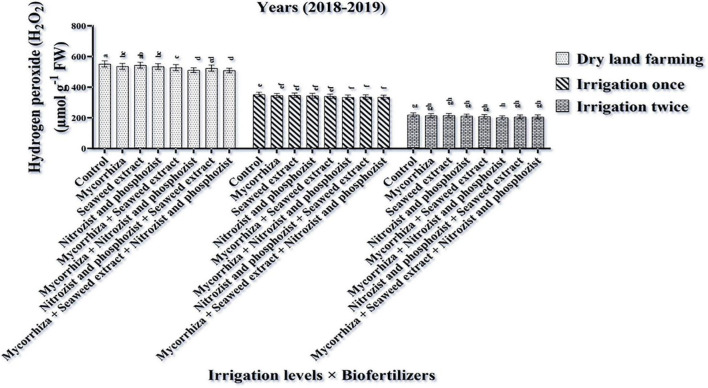
Mean comparisons of the dual interaction effect of irrigation × biofertilizers on hydrogen peroxide content in wheat plant in 2018–2019. Means in each column followed by similar letter(s) are not significantly different at 1% probability level by the LSD test.

Based on the results of comparing the mean of the first season of the experiment (Table 6), the lowest amount of H_2_O_2_ with an average of 276.69 μmol g^–1^ FW belonged to the bio-fertilizers of Mycorrhiza + Nitrozist and Phosphozist + Seaweed extract. All combination bio-fertilizer treatments had less H_2_O_2_ than the control treatment. Comparing the average effect of irrigation × bio-fertilizers explained (Figure 6) that in all levels of application or non-application of bio-fertilizers, the highest amount of H_2_O_2_ was obtained in the absence of irrigation. Supplementary irrigation, especially two-time irrigation, significantly reduced the amount of this trait in wheat.

### Proline Content

According to the results of ANOVA of the data obtained from the first and second seasons of the experiment, the simple effects of irrigation, cultivar, and bio-fertilizer treatments had a significant (*p* ≤ 0.01) on plant proline content (Supplementary Tables 2, 3). In both the experiment seasons, only the dual interactions of irrigation × cultivar and irrigation × bio-fertilizer were significant at a 1% probability level (Supplementary Tables 2, 3). The results of comparing the mean of the dual interaction of cultivar × irrigation in both seasons of the experiment showed that the highest amounts of proline were obtained for both cultivars (Sardari and Sirvan) in dry land conditions, and supplementary irrigation significantly reduced the plant proline content (Tables 7, 8). In both the seasons of the experiment and at all irrigation levels, the highest amount of proline belonged to the Sardari cultivar. In some cases, the difference between the two cultivars was insignificant (Tables 7, 8).

**TABLE 7:** Comparison of the mean of the dual interaction of cultivar and irrigation on some studied traits of wheat in 2017–2018.

Irrigation level × Cultivars	Proline (mg g^–1^ FW)	Chlorophyll a (mg g^–1^ FW)	Total chlorophyll (a + b) (mg g^–1^ FW)
Dry land farming × Sardari	1.4577 ± 0.0233^a^	1.1953 ± 0.0479^e^	1.8146 ± 0.0210^f^
Dry land farming × Sirvan	0.9054 ± 0.0175^b^	1.4620 ± 0.0479^d^	2.1541 ± 0.0290^e^
One-time irrigation × Sardari	0.6404 ± 0.0036^c^	1.9707 ± 0.0481^c^	2.8965 ± 0.0355^d^
One-time irrigation × Sirvan	0.5563 ± 0.0050^cd^	2.1675 ± 0.0478^c^	3.2780 ± 0.0363^c^
Two-time irrigation × Sardari	0.4274 ± 0.0090^de^	2.6569 ± 0.0478^b^	3.8267 ± 0.0586^b^
Two-time irrigation × Sirvan	0.2953 ± 0.0087^e^	3.0513 ± 0.0481^a^	4.3884 ± 0.0734^a^

*The data are means of four replicates ± SE. Means in each column followed by similar letter(s) are not significantly different at a 1% probability level by the LSD test.*

**TABLE 8:** Mean comparisons of the dual interactions of irrigation and cultivars on some traits in wheat plant in 2018–2019.

Treat (cultivars × irrigation)	Proline content (mg g^–1^ FW)	Chlorophyll a (mg g^–1^ FW)	Chlorophyll b (mg g^–1^ FW)
Sardari × Dry land farming	1.6143 ± 0.0172^a^	0.8879 ± 0.0481^d^	0.4984 ± 0.0320^c^
Sirvan × Dry land farming	1.1210 ± 0.0192^b^	1.2863 ± 0.0447^c^	0.5554 ± 0.0255^bc^
Sardari × One-time irrigation	0.6702 ± 0.0055^c^	1.7590 ± 0.0143^b^	0.7429 ± 0.0384^bc^
Sirvan × One-time irrigation	0.5844 ± 0.0058^cd^	1.9209 ± 0.0253^b^	0.8902 ± 0.0214^bc^
Sardari × Two-time irrigation	0.4793 ± 0.0072^d^	2.3944 ± 0.0226^a^	0.9256 ± 0.0392^b^
Sirvan × Two-time irrigation	0.3287 ± 0.0047^e^	2.4481 ± 0.0254^a^	1.3366 ± 0.0330^a^

*The data are means of four replicates ± SE. Means in each column followed by similar letter(s) are not significantly different at 1% probability level by the LSD test. MSI, cell membrane stability index.*

**FIGURE 7:**
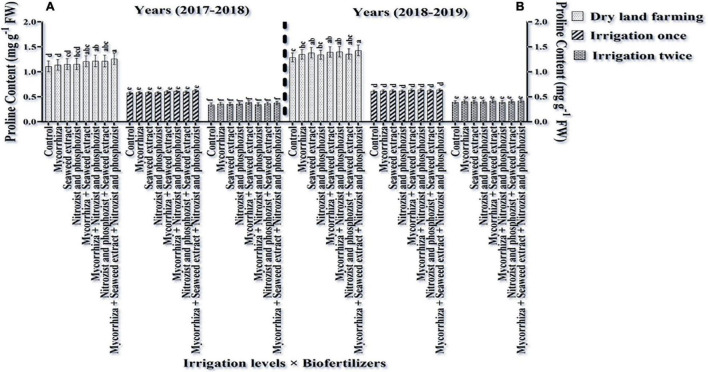
Mean comparisons of the dual interaction effect of irrigation × biofertilizers on proline content of wheat plant in two seasons of experiment **(A,B)** (2017–2018 and 2018–2019). Means in each column followed by similar letter(s) are not significantly different at 1% probability level by the LSD test.

Comparing the mean of the interaction of irrigation × bio-fertilizers in the first and second seasons of the experiment displayed that at all levels of application or non-application of bio-fertilizers, the highest proline content was obtained in the absence of irrigation. Supplementary irrigation, especially two-time irrigations, caused a significant reduction of the amino acid (proline) in wheat (Figure 7). Also, two seasons of research results showed that in dry land conditions, the application of bio-fertilizers significantly increased plant proline content. This increase as compared to the treatment of control without fertilizer application in the first and second seasons was 13.66 and 10.78% more, respectively (Figure 7).

### Content of Alcohol- and Water-Soluble Carbohydrates

According to the results of the experiment obtained by analyzing the data from the first two seasons, the irrigation, cultivar, and bio-fertilizer had a significant effect on the content of soluble carbohydrates of wheat. Also, in the second year, the dual interaction of bio-fertilizer × cultivar on the amount of alcohol-soluble carbohydrates was significant (*p* ≤ 0.05) (Supplementary Tables 2, 3). The results showed that complementary irrigation significantly decreased the content of alcohol- and water-soluble carbohydrates in both seasons (Tables 3, 9).

**TABLE 9:** Mean comparisons of simple main effects of irrigation levels on some traits in the wheat plant in 2018–2019.

Irrigation	ASC (mg g^–1^ dw)	WSC (mg g^–1^ dW)	Yield grain (kg ha^–1^)
Dry land farming	120.2900 ± 2.0654^a^	92.2072 ± 1.4403^a^	3317.0300 ± 54.1807^c^
One-time irrigation	80.6245 ± 1.5117^b^	73.2369 ± 1.4342^b^	5006.4100 ± 77.1675^b^
Two-time irrigation	53.0847 ± 0.7156^c^	44.2713 ± 1.0206^c^	5927.1900 ± 51.0526^a^

*The data are means of four replicates ± SE. Means in each column followed by similar letter(s) are not significantly different at 1% probability level by the LSD test. ASC, alcohol-soluble carbohydrate; WSC, water-soluble carbohydrates.*

**FIGURE 8:**
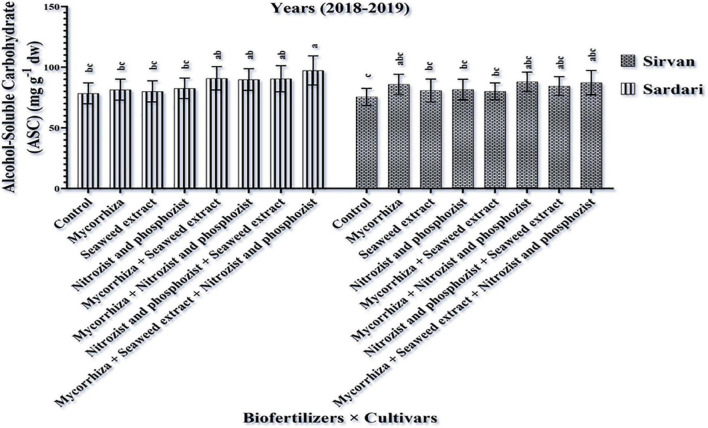
Mean comparisons of the dual interaction effect of biofertilizers × cultivars on alcohol-soluble carbohydrate content of wheat plant in 2018–2019. Means in each column followed by similar letter(s) are not significantly different at 1% probability level by the LSD test.

The simple effect of cultivar in the first season on the amount of alcohol-soluble carbohydrates in Sardari and Sirvan cultivars revealed (Tables 4, 5) that the amount of alcohol-soluble carbohydrates in the Sardari cultivar was equal to 75.01 mg g^–1^ dw, which was higher than the Sirvan cultivar with an average of 67.63 mg g^–1^ dw. In the study of the simple effect of bio-fertilizer treatments in the first year of the experiment, the lowest amount of alcohol-soluble carbohydrates (with an average of 63.17 mg g^–1^ dw) was allocated to the non-application of bio-fertilizers (control), whereas the highest amount of alcohol-soluble carbohydrates (with an average of 78.69 mg g^–1^ dw) was allocated to the application of Mycorrhiza + Nitrozist and Phosphozist + Seaweed extract bio-fertilizers (Table 6). In this research, the response of cultivars studied to bio-fertilizers application differed. The second-year results showed that the highest amount of alcohol-soluble carbohydrates in the Sardari cultivar was obtained from bio-fertilizers of Mycorrhiza + Nitrozist and Phosphozist + Seaweed extract (with an average of 97.30 mg g^–1^ dw). It increased by 24.04% compared to non-application bio-fertilizer treatment (with an average of 78.44 mg g^–1^ dw) (Figure 8). Except for the above case, no difference was observed in the Sardari cultivar between other treatments. In the Sirvan cultivar, although bio-fertilizers increased the number of alcohol-soluble carbohydrates compared to not using it, it did not make a significant difference.

In both seasons of the experiment, the Sirvan cultivar had the lowest water-soluble carbohydrates, 56.16 and 66.70, respectively. The Sardari cultivar with averages of 67.04 and 72.82 mg g^–1^ dw, respectively, had higher amounts of water-soluble carbohydrates. In comparison with the control treatment (non-application of bio-fertilizers), the results showed that Mycorrhiza + Nitrozist and Phosphozist + Seaweed extract bio-fertilizers increased the content of water-soluble carbohydrates by 30.75 and 17.81%, respectively, in the first and second seasons of research (Tables 6, 10).

**TABLE 10:** Mean comparisons of simple effects of bio-fertilizers on some traits in the wheat plant in 2018–2019.

Bio-fertilizers	WSC (mg g^–1^ dW)	Chlorophyll b (mg g^–1^ FW)	Yield grain (kg ha^–1^)
Control	63.8699 ± 4.6242^d^	0.7424 ± 0.5611^d^	4602.9200 ± 250.2100^c^
Mycorrhiza	69.9802 ± 4.5089^abc^	0.8135 ± 0.0682^bcd^	4685.4200 ± 244.2220^bc^
Seaweed extract	66.5757 ± 4.7740^cd^	0.8041 ± 0.0630^bcd^	4698.3300 ± 250.8700^bc^
Nitrozist and Phosphozist	67.9062 ± 4.3040^bcd^	0.8175 ± 0.0630^abcd^	4710.8300 ± 250.7830^bc^
Mycorrhiza + Seaweed extract	70.9439 ± 4.5457^abc^	0.8801 ± 0.0682^ab^	4790.4200 ± 248.1860^ab^
Mycorrhiza + Nitrozist and Phosphozist	73.0280 ± 4.8541^ab^	0.8588 ± 0.0700^abc^	4758.3900 ± 246.7500^b^
Nitrozist and Phosphozist + Seaweed extract	71.6938 ± 4.4307^abc^	0.7737 ± 0.0721^cd^	4802.5000 ± 243.8610^ab^
Mycorrhiza + Seaweed extract + Nitrozist and Phosphozist	75.2432 ± 4.5689^a^	0.9083 ± 0.0782^a^	4923.3300 ± 239.6900^a^

*The data are means of four replicates ± SE. Means in each column followed by similar letter(s) are not significantly different at 1% probability level by the LSD test. MSI, cell membrane stability index.*

### Cell Membrane Stability Index

**FIGURE 9:**
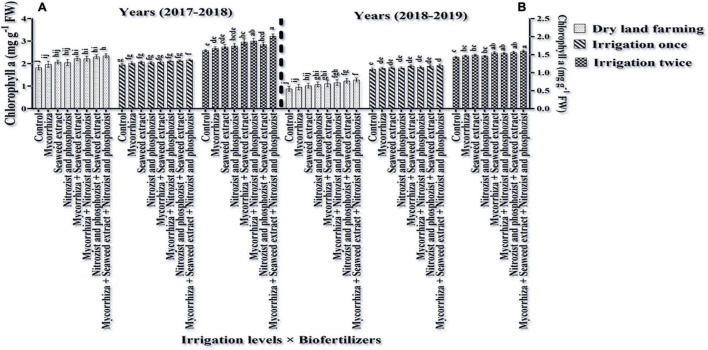
Mean comparisons of the dual interaction effect of irrigation × biofertilizers on Chlorophyll a content of wheat plant in two seasons of experiment **(A,B)** (2017–2018 and 2018–2019). Means in each column followed by similar letter(s) are not significantly different at 1% probability level by the LSD test.

According to the ANOVA of data of the first- and second seasons of the experiment, the main effects of irrigation, cultivar, and bio-fertilizer had a significant effect on the MSI at a 1% probability level (Supplementary Tables 2, 3). Based on the results of ANOVA in both seasons, only the dual interaction of irrigation × bio-fertilizer was significant at 5% probability level (Supplementary Tables 2, 3). As shown in Tables 4, 5, in the first and second seasons of the study, the MSI in Sardari cultivar (72.43 and 67.40%, respectively) compared to the Sirvan cultivar (61.82 and 56.93, respectively) was significantly higher. The mean of the data in the first and second seasons of the experiment illustrated (Figure 9) that supplementary irrigation, especially two-time irrigations, significantly increased the membrane stability index in both seasons.

**FIGURE 10:**
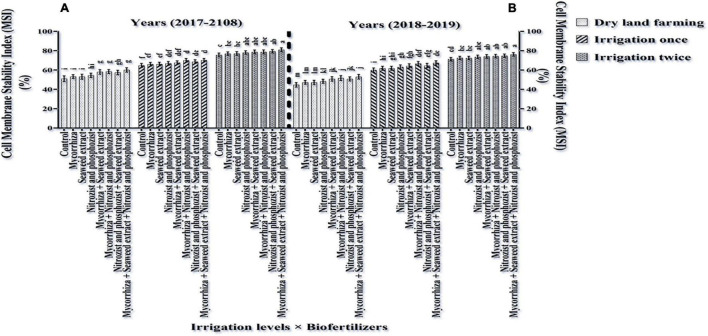
Mean comparisons of the dual interaction effect of irrigation × biofertilizers on cell MSI in wheat plant in two seasons of experiment **(A,B)** (2017–2018 and 2018–2019). Means in each column followed by similar letter(s) are not significantly different at 1% probability level by the LSD test.

The results showed that all fertilizer treatments increased the wheat membrane stability index under two-time irrigation. Wheat treated with bio-fertilizers Mycorrhiza + Nitrozist and Phosphozist + Seaweed extract had the highest membrane stability index in the first and second seasons (80.97 and 76.12%, respectively) among fertilizer treatments (Figure 10).

### Chlorophylls a, b, Total (a + b), and Carotenoids

As shown in Tables 3, 4, in the first and second seasons of the experiment, the simple effects of irrigation, cultivar, and bio-fertilizer on chlorophyll “a,” chlorophyll “b,” total chlorophyll (a + b), and carotenoids were significant at 1% probability level. The interaction of irrigation × cultivar was significant in the first season for chlorophyll a and total chlorophyll (a + b) and in the second season for chlorophyll “a” and chlorophyll “b” (Supplementary Tables 2, 3). Also, the interaction of irrigation × bio-fertilizer in the first and second seasons of the experiment was significant on chlorophyll “a,” total chlorophyll, and carotenoid traits. The triple interaction of irrigation × cultivar × bio-fertilizer on the chlorophyll “a” trait was significant (Supplementary Tables 2, 3).

**FIGURE 11:**
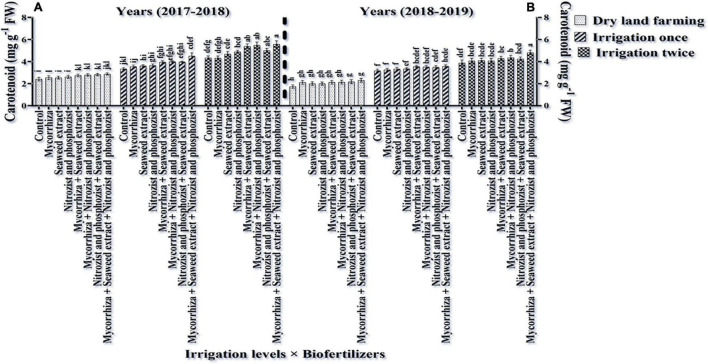
Mean comparisons of the dual interaction effect of irrigation × biofertilizers on carotenoids content of wheat plant in two seasons of experiment **(A,B)** (2017–2018 and 2018–2019). Means in each column followed by similar letter(s) are not significantly different at 1% probability level by the LSD test.

**FIGURE 12:**
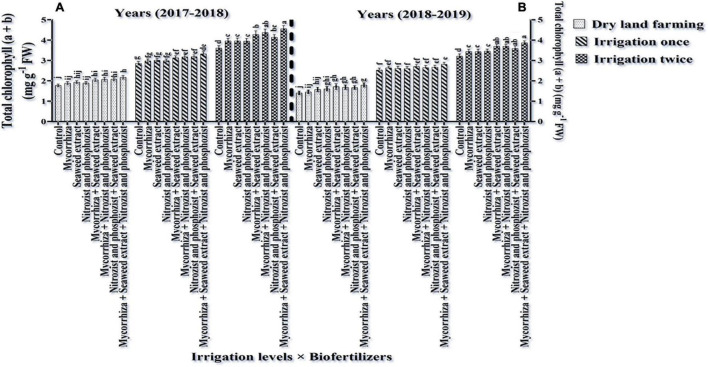
Mean comparisons of the dual interaction effect of irrigation × biofertilizers on total chlorophyll content of wheat plant in two seasons of experiment **(A,B)** (2017–2018 and 2018–2019). Means in each column followed by similar letter(s) are not significantly different at 1% probability level by the LSD test.

Comparing the mean illustrates that in both seasons of experimentation, supplementary irrigation (especially two-time irrigations) and application of bio-fertilizers improved the content of chlorophyll a, total, and carotenoids in wheat. Plants treated with two-time irrigation and bio-fertilizers, especially Mycorrhiza + Nitrozist and Phosphozist + Seaweed extract, had the highest amounts of chlorophyll “a,” total chlorophyll (a + b), and carotenoids compared to the control (non-application of bio-fertilizers and dry land) (Figures 9, 11, 12).

The results related to the effect of using bio-fertilizers in the first and second seasons of the study confirmed that the application of bio-fertilizers increased the amount of chlorophyll “b” compared to the control treatment (non-application of bio-fertilizers), and the greatest effect was related to the simultaneous use of Mycorrhiza + Nitrozist and Phosphozist + Seaweed extract bio-fertilizers, which caused a significant increase in chlorophyll “b” content compared to non-application of fertilizer and most fertilizer treatments containing one type of bio-fertilizer (Tables 6, 10). The results of plant carotenoid content showed that the Sirvan cultivar in the first and second seasons of the experiment had a higher carotenoid content (4.02 and 3.51 mg g^–1^ FW, respectively) than the Sardari cultivar (3.62 and 2.97 mg g^–1^ FW) (Table 4).

### Grain Yield

Based on the ANOVA of the data of the first and second seasons of the experiment (Supplementary Tables 2, 3), wheat grain yield was affected by the main effects of irrigation, cultivar, and bio-fertilizer at a 1% probability level. According to the results (Tables 3, 9), irrigation increased grain yield so that in the first and second season of the experiment, treatment of two-time irrigations (with an average of 6,250.38 and 5,927.197 kg/ha) compared to non-irrigated treatment (dry land) (with an average of 3481.88 and 3317.03 kg/ha) increased grain yield about 79.51 and 78/69%, respectively. In the first and second seasons of the experiment, the Sirvan cultivar with 5,447.34 and 5,055.08 kg/ha produced a higher grain yield than the Sardari cultivars with 4,458.34 and 4,437.94 kg/ha (Tables 4, 5).

According to the mean comparisons, it was observed that the application of bio-fertilizers in the first and second seasons of the experiment improved wheat grain yield. In the first and second seasons of the experiment, maximum grain yield was obtained by applying bio-fertilizers of Mycorrhiza + Nitrozist and Phosphozist + Seaweed extract (with an average of 5,226.25 and 4,923.33 kg/ha, respectively). The lowest values were obtained in non-application of bio-fertilizer conditions (with an average of 4,837.08 and 4,602.92 kg/ha, respectively) (Tables 6, 10).

## Discussion

Drought is one of the most important abiotic stresses that limit the growth and yield of different plants. In recent decades, climate changes caused by global warming have increased the severity of this stress (Naderi et al., 2020). Finding solutions to reduce the effects of this stress on the yield of strategic crops, such as wheat, can be very important and influential. Complementary irrigation, beneficial soil microorganisms, and beneficial organic matter are the solutions that have been studied. Based on the data obtained from this study (Figure 1), the two-time irrigation treatments with bio-fertilizers (Mycorrhiza + Nitrozist and Phosphozist + Seaweed extract) application had a positive effect on root volume as compared to the control. We believe that inoculation with bio-fertilizers (Figure 1) is an effective strategy for expanding and greater volume of roots under stress. Perhaps, one of the reasons for the increase in root volume of wheat is the ability of *Enterobacter cloacae* and *Serratia marcescens* strains to produce exopolysaccharides (EPSs), which is due to the fibrous material produced by these strains and helps them to firmly stick to the root surface even in the lack of water and cause more root expansion and volume. In addition to indicating the absence of antagonistic properties between mycorrhiza fungi and bacteria in Nitrozist and Phosphozist bio-fertilizers, this result also indicated that mycorrhiza fungi and bacteria in Nitrozist and Phosphozist bio-fertilizers acted in harmony, increasing root volume. In accordance with our results, Narimani et al. (2018) in their research stated that root volume and dry root weight in irrigation treatment in the booting stage, 82.9 and 59.2% and in the flowering stage, 7.2 and 55.7% of the treatment without irrigation, respectively (dry land cultivation), was more. As per Figure 2 of this study, it can be concluded that the Sardari cultivar has allocated more dry matter to the roots and more photosynthetic materials for root production. However, more root volume allocation harms reproductive growth and yield. Nevertheless, this strategy is an important feature of drought-resistant cultivars for plant resistance to drought stress conditions.

**FIGURE 13:**
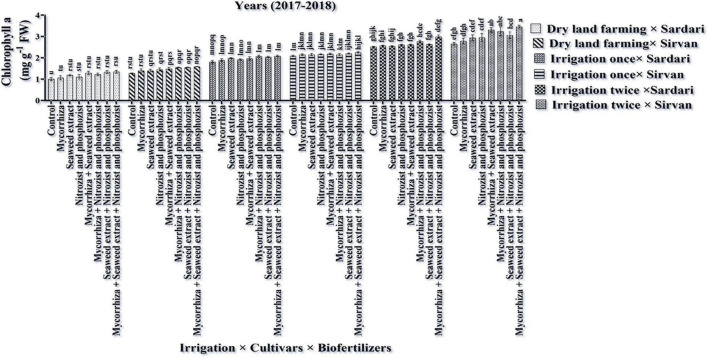
Mean comparisons of triple interaction effect of irrigation × cultivars × biofertilizers on Chlorophyll a content of wheat plant in 2017–2018. Means in each column followed by similar letter(s) are not significantly different at 1% probability level by the LSD test.

The results of the first year of the experiment revealed that the highest amount of chlorophyll “a” (3.46 mg g^–1^ FW) was obtained in the Sirvan cultivar treated with Mycorrhiza + Nitrozist and Phosphozist + Seaweed extract bio-fertilizers and under two-time irrigation conditions (Figure 13). The lowest amount of chlorophyll “a” (1.004 mg g^–1^ FW) was obtained in the Sardari cultivar without bio-fertilizers and under dry land conditions (Figure 13). The lack of irrigation and drought stress caused a significant decrease in chlorophyll “a” content, especially in the Sardari cultivar. The main effects of the factors revealed that the Sirvan cultivar had the highest amount of chlorophyll “a” and total chlorophyll (a + b) (2.23 and 2.81 mg g^–1^ FW, respectively) in the second season of the experiment. In comparison, the Sardari cultivar had the lowest amount of chlorophyll “a” and total chlorophyll (a + b) (1.94 and 2.40 mg g^–1^ FW, respectively) (Table 5). Also, in the first season of the experiment, the highest amount of chlorophyll “b” (1.25 mg g^–1^ FW) was obtained under two-time irrigation conditions. In comparison, the lowest amount of chlorophyll “b” (0.66 mg g^–1^ FW) was obtained under dry land conditions (control), indicating that the two-time irrigation treatments increased chlorophyll “b” by 91.17% when compared to the control treatment (dry land) (Table 3). In the first year of the experiment, the amount of chlorophyll “b” in the Sirvan cultivar (1.05 mg g^–1^ FW) was higher than in the Sardari cultivar (0.91 mg g^–1^ FW). Also, the results displayed that by reducing water consumption in the first season of the experiment, the total chlorophyll and chlorophyll “a” and in the second season, chlorophylls “a” and “b” in Sardari and Sirvan cultivars were decreased. This decrease was more in Sardari cultivar than in Sirvan cultivar (Table 7).

Naseri et al. (2020), in a study on Saji and cross-Sabalan cultivars of wheat, stated that the use of bio-fertilizers containing bacteria and fungi *Glomus mossea*e, because of their ability to improve root characteristics (root volume, root area, root density, and root accumulation), increased plant water content and consequently improved the growth characteristics of the plant. Therefore, it was shown that dry land wheat cultivars, including Saji and cross-Sabalan in dry land conditions, had more root length and volume compared to irrigated cultivars.

The findings (Table 3) show that two-time supplementary watering enhanced wheat root colonization. Azarmi Atajan et al. (2019) found that limiting water consumption reduced wheat root colonization. So, at 25% field capacity, this parameter’s value is reduced by 29% compared to full irrigation. Drought stress reduced spore germination and hypha growth, resulting in reduced root colonization. They also found that inoculating wheat seeds with plant growth-promoting bacteria improved root colonization percentages at various watering levels. Our study revealed that bio-fertilizers (Mycorrhiza + Nitrozist and Phosphozist + Seaweed extract) improved colonization (Figure 3). However, its combination application raised the percentage of colonization the most, perhaps because of enhanced water and mineral absorption. Pellegrino et al. (2020) reported that inoculating wheat with AMF enhanced root colonization. Elgharably and Nafady (2021) found that AMF inoculating wheat improved root colonization and nutrient absorption. More nitrogen and phosphorus absorptions by mycorrhiza fungi and phosphate-soluble and nitrogen-fixing bacteria increase root colonization and wheat stress tolerance, especially in enough water conditions.

The Sardari cultivar has a higher root volume (Figure 1) and better water retention capacity in the soil (Tables 4, 5), so it has a higher RWC. Increasing the RWC of the leaves indicates the plant’s resistance and internal stability to environmental stresses. In accordance with this study findings, Keikha et al. (2019) found that water deficit lowered RWC in wheat plants. Mamnabi et al. (2020) proposed that RWC variability across genotypes is related to genetic capacity for rhizosphere water uptake and root depth extension to access lower soil horizons for moisture extraction.

According to our study, bio-fertilizers (Mycorrhiza + Nitrozist and Phosphozist + Seaweed extract) enhance resistance and drought tolerance (Figures 4, 5). It enhances RWC in both irrigated and dry land environments in both cultivars (Sardari and Sirvan) (Figures 4, 5). Bio-fertilizers in wheat have improved root volume (Figure 1), osmotic regulation ability, and better water absorption and retention in the plant, which naturally results in greater RWC and lower WSD in these treated plants. According to Rashid et al. (2021), during drought stress, wheat plants inoculated with bio-fertilizers containing PGPR showed a higher concentration of compatible osmolytes (particularly, proline and soluble carbohydrates) in the cytosol. These key responses to stress raised the inoculated plant’s RWC and helped to maintain its turgor pressure. According to Boutasknit et al. (2021), mycorrhiza increased stomatal conductance relative to inoculated plants during drought stress. Auge et al. (2016) found that mycorrhiza fungus inoculation enhanced stomatal conductance relative to uninoculated plants. In fact, increasing the stomatal conductance can be attributed to higher phosphorus concentrations. According to Meddich et al. (2015), plants treated with mycorrhiza arbuscular fungus have larger pore conductance and better osmotic regulation. Mycorrhiza affects the plant’s hormonal balance, which regulates stomatal function under water stress (Miransari et al., 2014; Chitarra et al., 2016). In research on corn, it was reported that increasing soil moisture and inoculation of seeds with mycorrhiza fungi increased the percentage of plant RWC (Majidi and Amiri, 2020). Begum et al. (2021) found that plants treated with bio-fertilizers containing bacteria and AMF had the most excellent RWC levels. The formation of stable aggregates (which enhance climatic permeation), access of fungal hyphae to larger volumes of soil, enhancing water absorption capacity in plants, and increased gene expression are all factors that can help to regulate root permeability to water and improve plant water flow.

The reduced WSD rate in the Sardari cultivar (Tables 4, 5) is connected to higher root volume (Figure 2) and better water absorption and retention. The role of hyphae in absorbing and delivering water to the host plant’s root may be one of the reasons for mycorrhiza fungus increasing leaf RWC. The changing cycle of WSD under drought stress is utilized to determine the plant’s water status. The influence of stress (dry land) and irrigation (one-time irrigation and two-time irrigation) on WSD shows that WSD increases with stress. This research found that resistance to WSD was higher in the Sardari cultivar than in the Sirvan cultivar.

The findings showed that two-time irrigations in dry land conditions reduced H_2_O_2_ levels (Table 3). The increased root volume under supplementary irrigation (Figure 1), along with the plant’s improved water status (Table 3 and Figures 4, 5), lowered the formation of reactive oxygen species (H_2_O_2_). According to Abdi et al. (2021), water limitation caused the highest electrolyte leakage, H_2_O_2_, and MDA in wheat plants (Waszczak et al., 2018; Farooq et al., 2019). Plants create ROS, such as H_2_O_2_, to combat abiotic stresses. Drought-induced H_2_O_2_ generation damages membranes and disrupts plant cell redox control (Benaffari et al., 2022). The Sirvan cultivar contained higher H_2_O_2_ than the Sardari (Tables 4, 5). This is because Sardari has a more root volume (Figure 1), better osmotic control (Tables 4, 5, 7), and hence better water status (Tables 4, 5) than Sirvan. Using bio-fertilizers in one-time irrigation treatments decreased the plant’s H_2_O_2_ concentration, particularly in dry land conditions. Using bio-fertilizers (Mycorrhiza + Nitrozist and Phosphozist + Seaweed extract) in dry land conditions might reduce the H_2_O_2_ levels due to their ability to increase nutrient and water absorption (Figure 6). So, bio-fertilizers reduced H_2_O_2_ and improved drought stress tolerance. Adrees et al. (2020) found that under drought stress, non-mycorrhizal plants accumulated more H_2_O_2_ than mycorrhiza plants. AMF protects mitochondrial and chloroplast electron transporters by fine-tuning antioxidant components that eliminate H_2_O_2_ (Mona et al., 2017). According to Benaffari et al. (2022), inoculating quinoa with mycorrhiza improves the antioxidant system and increases the activity of antioxidant enzymes in quinoa organs. Antioxidant enzymes and metabolites help to remove ROS and reduce oxidative stress in plant cells. Trivedi et al. (2018) found that applying seaweed extract to maize leaves reduced MDA, superoxide radicals, and H_2_O_2_ levels.

Plants use osmotic control to maintain cell turgor pressure under drought stress by accumulating soluble compounds at low water potentials. So, proline is a cellular regulator in drought. Proline also protects membranes and increases membrane stability index (Tables 4, 5). It also reduces ROS free radicals and improves plant stress tolerance. The above results were consistent with the results of other researchers in this regard, so Aqcheh Lee et al. (2020) stated that under drought stress conditions, the proline content of wheat sharply increased. Proline is the most significant amino acid for stress. Proline is an osmotic protector, but it also regulates cellular balance (Podlesakova et al., 2019). Because one of the mechanisms for proline formation is glutamate, greater soluble sugar production increases glutamate production, and proline synthesis increases (Alikhani and Mahmudi Zarandi, 2019). Figure 7 shows that Mycorrhiza + Nitrozist and Phosphozist + Seaweed extract enhanced proline levels. The non-bio-fertilizer control treatment contained the least proline, which may be due to improved nutritional and aqueous conditions and better osmotic management, which enhanced growth and photosynthesis. This indicates that bio-fertilizers may help to increase drought resistance in the host plant. Increasing the number of physiological traits, such as proline, in bio-fertilizers reduces the effects of dehydration in dry land conditions, increasing plant growth. An increase in phytohormone production, such as indole acetic acid, gibberellins, and cytokinins, was observed by Galindo et al. (2019). Proline is a primary source of energy and nitrogen for metabolic activities of the plant under drought stress when inoculated with PGPR-containing bio-fertilizers (Xia et al., 2020; Rashid et al., 2021). Ghaffarizadeh et al. (2016), in a study on the wheat plant, showed that the highest amount of proline was observed in the treatment of 20% algae fertilizer and the lowest in the treatment of 10% Seaweed extract fertilizer. In wheat plants treated with seaweed extract, proline, soluble carbohydrates, and amino acids function as the osmotic protectors (Patel et al., 2018). Polyamines are low molecular weight molecules that increase in plant cells in response to environmental stress (Chen et al., 2019). Mycorrhiza fungi improve plant osmotic regulation by accumulating substances, such as proline and soluble carbohydrates. The coexistence of arbuscular mycorrhiza fungus with plant roots helps plants grow in drought-stressed areas (Tanveer et al., 2019).

In both seasons of the experiment, supplemental irrigation (two-time irrigations) lowered the amount of soluble carbohydrates in alcohol and water (Tables 5, 6). According to Dehghan et al. (2020), stressed wheat leaves have the most soluble carbohydrates in the flowering stage. The lowest amount of soluble carbohydrates was obtained in fully irrigated plants. Osmotic regulation is one of the mechanisms that may maintain cell turgidity and related processes at low water potentials by accumulating soluble substances in cells. In plants’ awns and shoots, low molecular weight organic compounds, such as soluble sugars and free amino acids, operate as osmotic regulators (Rahimi et al., 2019). Based on Tables 4, 5, the Sardari cultivar has more alcohol-soluble carbohydrates than Sirvan. Table 6 and Figure 8 of this research indicated that the Sardari cultivar showed a better response to bio-fertilizers. As shown in Tables 6, 10, the high concentration of soluble carbohydrates in alcohol and water in plants inoculated with bio-fertilizers of Mycorrhiza + Nitrozist and Phosphozist + Seaweed extract can increase plant growth through direct (uptake and transfer of soluble nitrogen) and indirect (secretion of organic compounds and soil insoluble nitrogen conversion to solution-phase and then transfer) mechanisms. Rashid et al. (2021) found that inoculating wheat plants with bio-fertilizers containing insoluble phosphate solubilizing and nitrogen-fixing bacteria increased the amount of soluble sugar and the activity of various ROS-removing enzymes but also the growth and photosynthetic efficiency of inoculated wheat plants. This is a protective reaction and a reason to develop abiotic stress tolerance. For water absorption, plants accumulate adaptive osmolytes, such as soluble sugars and proline (Tani et al., 2019). Using individual bio-fertilizers (Mycorrhiza, *Pseudomonas*, and Mycorrhiza with *Pseudomonas*) increased proline content and soluble sugars compared to not using them (Aghcheli et al., 2019).

The Sardari cultivar has a better membrane stability index than the Sirvan cultivar (Tables 4, 5). Because Sardari has more root volume (Figure 2), more RWC content (Tables 4, 5), better osmotic regulation *via* osmolyte accumulation (Tables 4, 5, 7, 8 and Figure 10), and less oxidative stress than Sirvan, it is more adapted to water shortage conditions than Sirvan. The combination of Mycorrhiza + Nitrozist and Phosphozist + Seaweed extract enhanced membrane stability index (Figure 10) compared to dry land treatments. By reducing H_2_O_2_ level (Figure 6) and oxidative stress produced by ROS, supplementary irrigation decreased cell membrane damage and enhanced membrane stability. In research, Motazedi et al. (2019) found that supplementary watering increased MSI by 12.7% compared to dry land plants. According to El-hady et al. (2018), severe water restriction induces cellular oxidative damage and significant lipid peroxidation, reducing membrane stability and increasing cell electrolyte leakage. Because bio-fertilizers reduce oxidative stress caused by drought, membrane permeability is reduced. It is believed that increasing the MSI with bio-fertilizers is due to their positive effect on root volume (Figures 1, 2) and osmotic regulation capacity (Tables 6, 10), which improved the plant’s capacity for water uptake and retention (Tables 6, 10 and Figures 4, 5). This subject lowered the amount of H_2_O_2_ (Figure 6) and perhaps other reactive oxygen species that damage plant cell membranes. Drought tolerance mechanisms in quinoa plants inoculated with MA fungus help to limit ROS and maintain antioxidant levels, according to the study by Benaffari et al. (2022). The AsA-GSH cycle contains antioxidant enzymes and electron donors. Trivedi et al. (2018) reported that seaweed extract enhanced photosynthetic rate, decreasing antioxidant enzyme levels in foliar maize plants. According to Mamnabi et al. (2020), bio-fertilizers increase MSI and reduce plant membrane lipid peroxidation.

Bio-fertilizers Mycorrhiza + Nitrozist and Phosphozist + Seaweed extract and supplemental irrigation, in particular, “two irrigation” enhanced chlorophyll a, total chlorophyll (a + b), and carotenoid content (Figures 9, 11, 12). This impact of bio-fertilizers is related to longer leaf longevity, more photosynthetic pigments, and improved access to water and nutrients. The Sirvan and Sardari cultivars showed higher chlorophyll “a” under two-time irrigation and Mycorrhiza + Nitrozist and Phosphozist + Seaweed extract application (Figure 13). Layek et al. (2018) reported that 10% aqueous extract of brown algae and 34.5 kg/ha nitrogen consumption caused a significant increase in leaf area, photosynthetic pigments, and soluble carbohydrates cytoplasmic proteins in rice. Koobaz et al. (2020) found that drought reduced wheat’s chlorophyll “a,” “b,” and carotenoid concentration. Oxidative damage to chloroplast membrane lipids and proteins and plant photosynthetic pigments may cause decreased chlorophyll “a” and “b” levels (Koobaz et al., 2020). Dadashzadeh et al. (2019) observed that increasing photosynthesis and antioxidant activity of the plant enhanced the content of chlorophylls a, b, total, and carotenoids in the flag leaf of barley. They found that inoculating seeds with *Azospirillum* and AMF bio-fertilizer produced the maximum level of chlorophyll a, b, total, and carotenoids. The lowest levels of these traits were achieved without supplemental watering and without inoculating seedlings with the mentioned microorganisms. Increasing carotenoid content in bio-fertilizers containing *Azospirillum* and *Azotobacter* bacteria revealed their dual function in preserving carotenoids and reducing ROS oxidation (Yaghini et al., 2020). The research indicated that seaweed extract increased the concentration of chlorophylls “a,” “b,” and carotenoids in wheat (Shahbazi et al., 2015). Mycorrhiza + Nitrozist and Phosphozist + Seaweed extract bio-fertilizers stabilized photosynthesis and increased chlorophyll ratio during the stress period. This research found that the Sirvan cultivar has higher carotenoids than the Sardari (Table 4). Carotenoid pigments used in photosynthesis may be utilized as antioxidants to reduce the effects of free radicals on plants and thereby reduce oxidative stress during adaptation.

Supplemental irrigation can help to increase wheat yield by reducing the effects of water scarcity (Tables 3, 9). Drought stress reduces membrane stability and osmotic regulation, impairs water transport, and damages the plant photosynthetic machinery (Sanjari et al., 2021). The Sardari cultivar had larger root volume, RWC, proline content, MSI, and less H_2_O_2_ than the Sirvan cultivar, but had a lower yield. The Sirvan cultivar outperforms the Sardari cultivar in terms of grain yield due to genetic differences between the two cultivars. Plants need energy and photosynthetic resources to make and accumulate osmotic compounds. This reduces dry matter accumulation and grain yield while increasing plant stress tolerance. The decreased grain production of Sardari dry-land cultivar under drought stress may be due to early leaf aging and reduced green leaf area for photosynthesis in reproductive plants (Rozo-Ortega et al., 2021; Snowdon et al., 2021). The increased chlorophyll “a,” “b,” total, and carotenoid contents, and colonization percentage of this cultivar, in addition to helping to maintain green leaf area, have increased the ability of photosynthesis and production in this irrigated cultivar (Tables 4, 5). Nitrogen-fixing bacteria in bio-fertilizers improved chlorophyll production, enhancing crop yield (Tables 6, 10). The positive effect of phosphate-solubilizing bacteria and mycorrhiza fungi on percentage colonization, yield, and yield components of wheat, especially in water-stressed conditions, can be attributed to bacteria producing auxin, organic acids, siderophore, and mycorrhiza fungi inoculating roots, increasing soil volume and thus water and phosphorus absorption for the plant (Azarmi Atajan et al., 2019). According to wheat research, bio-fertilizers that contain growth-promoting bacteria (PGPR) could dissolve phosphate and potassium while boosting access to insoluble and inaccessible trace elements (Danish and Zafar-ul-Hye, 2019; Kour et al., 2020; Rashid et al., 2021). Mahato and Kafle. (2018) found that inoculating wheat seedlings with *Azotobacter* bio-fertilizer increased grain yield, total biomass, and biological yield. In the study, the co-existence of quinoa with arbuscular mycorrhiza fungi and physio-metabolic mechanisms due to the availability of nutrients in the soil makes the quinoa plant tolerant to drought stress. Rafi et al. (2021) stated that using growth stimulants such as Seaweed extract in drought stress conditions caused a significant increase in wheat grain yield compared to non-using these substances. In addition, growth stimulants such as seaweed extract compensated for drought stress. Seaweed extract significantly increased wheat grain yield compared to non-using these compounds under optimal irrigation conditions. In practical and experimental terms, mechanisms, such as increasing soil fertility and promoting plant growth, and physiological and biochemical properties of quinoa, continuously improved antioxidant enzyme defense and reduced the accumulation of oxidative stress markers (Benaffari et al., 2022). Genes associated with photosynthesis, nitrogen metabolism, polysaccharide synthesis, and signal transduction also influence ups and downs. These genes show how seaweed extract reduces drought in maize (Trivedi et al., 2021). Increasing root volume and root colonization percentage (Figures 1–3), improving plant water status (Figures 4, 5 and Table 10), and increasing photosynthetic pigments content (Figures 9, 11–13 and Tables 6, 10) improved plant photosynthesis. Increasing photosynthesis and assimilation increased grain yield.

The use of bio-fertilizers containing beneficial soil microorganisms increases their population, which has reduced due to unprincipled agricultural practices, excessive chemical use, and environmental stresses. Using these microbes can help to minimize drought stress in water-scarce areas by enhancing soil microbial activity, improving fertility, and stabilizing soil mineral components. These two options can help to fulfill the increasing food demand of the 21st century while protecting the environment and its privacy.

## Conclusion

According to this research, the use of additional irrigation and bio-fertilizers, particularly the simultaneous use of various bio-fertilizers, increased the water status of wheat. The application of bio-fertilizers in wheat due to increasing the population of growth-promoting microorganisms in the root zone of the plant and the positive effects of these microorganisms or compounds of these fertilizers caused an increase in root volume and osmotic regulation ability of the plant and better absorption and maintenance of water in the plant. Plant hydrogen peroxide content and oxidative stress caused by reactive oxygen species were reduced due to improved water status in plants treated with supplementary irrigation and bio-fertilizers, followed by reduced damage to cell membranes, macromolecule structure, and photosynthetic pigments, and finally improved plant yield. Sardari dry land cultivar had more root volume and better osmotic regulation through the osmolyte accumulation. As a result of the higher RWC, it had less H_2_O_2_ and higher MSI than the Sirvan irrigated cultivar under water-deficit conditions. Therefore, it showed better adaptation to water shortage stress. Despite the superiority of the Sardari cultivar over the Sirvan cultivar in most of the studied traits in the first and second seasons of the experiment, the Sirvan cultivar produced about 1,019 and 617.14 kg/ha, respectively, higher grain yield than the Sardari cultivar. The presence of more photosynthetic pigments in the Sirvan cultivar and its better photosynthetic power and yield potential compared to the Sardari cultivar may explain its superior yield. On the other hand, although the accumulation of higher amounts of osmolytes in the Sardari cultivar causes more resistance of this cultivar to stress conditions under low and moderate stress conditions, this increase in accumulation is associated with the consumption of nitrogen and photosynthetic material of plants and reduces dry matter accumulation and grain yield in this cultivar.

## Data Availability Statement

The original contributions presented in the study are included in the article/Supplementary Material. The datasets used and/or analyzed during the current study are available from the corresponding author on reasonable request.

## Author Contributions

YS: supervisor of students in the Ph.D. thesis project, designing farm experiments, managing laboratory works, guiding and helping with statistical analysis of data, and writing the manuscript. ZN: performing practical works in the farm and the laboratory, statistical analysis of data obtained from the experiment, writing the manuscript, and plotting the figures related to the manuscript. GM: advisor of students in the Ph.D. thesis project, guiding and helping with statistical analysis of data, and writing the manuscript. GH: advisor of students in the Ph.D. thesis project, guiding and helping with statistical analysis of data, and writing the manuscript.

## Conflict of Interest

The authors declare that the research was conducted in the absence of any commercial or financial relationships that could be construed as a potential conflict of interest.

## Publisher’s Note

All claims expressed in this article are solely those of the authors and do not necessarily represent those of their affiliated organizations, or those of the publisher, the editors and the reviewers. Any product that may be evaluated in this article, or claim that may be made by its manufacturer, is not guaranteed or endorsed by the publisher.

## Footnotes

1 EC has been used principally as a measure of soil salinity.
