# Supplementary material for: Soil Microorganisms and Seaweed Application With Supplementary Irrigation Improved Physiological Traits and Yield of Two Dryland Wheat Cultivars
Source: Front Plant Sci. 2022 Jun 1;13:855090. doi: 10.3389/fpls.2022.855090 (PMC9198557; doi:10.3389/fpls.2022.855090)
Supplement: Supplementary file 1 [file Data_Sheet_1.docx]

**Supporting tables**

**Supplementary Table S1 |** Combined analysis of variance of the effect of supplementary irrigation and bio-fertilizers application on studied traits of wheat in two seasons of experiment (2017-2018 and 2018-2019).

**Supplementary Table S2 |** Results of analysis of variance of studied traits of wheat cultivars under the influence of supplementary irrigation and bio-fertilizers levels in 2017-2018.

**Supplementary Table S3 |** Results of analysis of variance of studied traits of wheat cultivars under the influence of supplementary irrigation and bio-fertilizers levels in 2018-2019.

**Supplementary Table S1 |** Combined analysis of variance of the effect of supplementary irrigation and bio-fertilizers application on studied traits of wheat in two seasons of experiment (2017-2018 and 2018-2019).

|  | Mean square |  |
| --- | --- | --- |
| S.O.V. | **df** | **Root Volume (RV)** |
| Years | 1 | ns |
| (Repeat ×Y) | 6 | ns |
| Irrigation | 2 | ** |
| (Y× I) | 2 | ns |
| (Ea) | 12 | - |
| Cultivars | 1 | ns |
| (I × C) | 2 | ns |
| (Y × C) | 1 | ns |
| (Y × I × C) | 2 | ns |
| (Eb) | 18 | - |
| Biofertilizers | 7 | ** |
| (I × B) | 14 | ** |
| (C × B) | 7 | ** |
| (I × C × B) | 14 | ns |
| (Y × B) | 7 | ns |
| (Y × I × B) | 14 | ns |
| (Y × C × B) | 7 | ns |
| (Y× I × C × B) | 14 | ns |
| )Ec) | 252 | - |
| (%) C.V. | - | 11.3052 |

ns, *, ** non-significant, significant at *P* < 0.05 and *P* < 0.01, respectively

**Supplementary Table S2 |** Results of analysis of variance of studied traits of wheat cultivars under the influence of supplementary irrigation and bio-fertilizers levels in 2017-2018.

|  |  | Mean square | | | | | | |
| --- | --- | --- | --- | --- | --- | --- | --- | --- |
| S.O.V. | **df** | **PC** | **RWC** | **WSD** | **H_2_O_2_** | **Proline Content** | **ASC** | **WSC** |
| Repeat | 3 | ns | ns | ns | ns | ns | ns | ns |
| Irrigation (I) | 2 | ** | ** | ** | ** | ** | ** | ** |
| (Ea) | 6 | - | - | - | - | - | - | - |
| Cultivars (C) | 1 | ** | ** | ** | ** | ** | * | ** |
| (C ×I) | 2 | ns | ns | ns | ns | ** | ns | ns |
| (Eb) | 9 | - | - | - | - | - | - | - |
| Biofertilizers (B) | 7 | ** | ** | ** | ** | ** | ** | ** |
| (B × I) | 14 | ns | ** | ** | ns | ** | ns | ns |
| (B × C) | 7 | ns | ns | ns | ns | ns | ns | ns |
| (B × I × C) | 14 | ns | ns | ns | ns | ns | ns | ns |
| (Ec) | 126 | - | - | - | - | - | - | - |
| C.V.(%) | - | 3.3576 | 1.4586 | 5.5561 | 2.6094 | 4.8309 | 8.7461 | 9.3760 |

ns, *, ** non-significant, significant at *P* < 0.05 and *P* < 0.01, respectively. PC= Percentage of Colonization, RWC= Relative Water Content, WSD= Water Saturate Deficit, ASC= Alcohol-Soluble Carbohydrate, WSC= Water-Soluble Carbohydrates.

**Supplementary Table S2 |**

|  |  | Mean square | | | | | |
| --- | --- | --- | --- | --- | --- | --- | --- |
| S.O.V. | **df** | **MSI** | **Chlorophyll a** | **Chlorophyll b** | **Total chlorophyll (a + b)** | **Carotenoid** | **Grain Yield** |
| Repeat | 3 | ns | ns | ns | ns | * | ns |
| Irrigation (I) | 2 | ** | ** | ** | ** | ** | ** |
| (Ea) | 6 | - | - | - | - | - | - |
| Cultivars (C) | 1 | ** | ** | ** | ** | ** | ** |
| (C ×I) | 2 | ns | ns | ns | ** | ns | ns |
| (Eb) | 9 | - | - | - | - | - | - |
| Biofertilizers (B) | 7 | ** | ** | ** | ** | ** | ** |
| (B × I) | 14 | * | ** | ns | ** | ** | ns |
| (B × C) | 7 | ns | ns | ns | ns | ns | ns |
| (B × I × C) | 14 | ns | ns | ns | ns | ns | ns |
| (Ec) | 126 | - | - | - | - | - | - |
| C.V.(%) | - | 2.7748 | 5.6847 | 13.5208 | 4.7339 | 9.1976 | 4.1652 |

Continued

ns, *, ** non-significant, significant at *P* < 0.05 and *P* < 0.01, respectively. MSI= Cell Membrane Stability Index.

**Supplementary Table S3 |** Results of analysis of variance of studied traits of wheat cultivars under the influence of supplementary irrigation and biofertilizers levels in 2018-2019.

|  |  | Mean square | | | | | | |
| --- | --- | --- | --- | --- | --- | --- | --- | --- |
| S.O.V. | **df** | **PC** | **RWC** | **WSD** | **H_2_O_2_** | **Proline**  **Content** | **ASC** | **WSC** |
| Repeat | 3 | ns | ns | ns | ns | ns | ns | ns |
| Irrigation (I) | 2 | ** | ** | ** | ** | ** | ** | ** |
| (Ea) | 6 | - | - | - | - | - | - | - |
| Cultivars (C) | 1 | ** | ** | ** | ** | ** | * | * |
| (C ×I) | 2 | ns | ns | ns | ns | ** | ns | ns |
| (Eb) | 9 | - | - | - | - | - | - | - |
| Biofertilizers (B) | 7 | ** | ** | ** | ** | ** | ** | ** |
| (B × I) | 14 | ** | * | * | ** | ** | ns | ns |
| (B × C) | 7 | ns | ns | ns | ns | ns | * | ns |
| (B × I × C) | 14 | ns | ns | ns | ns | ns | ns | ns |
| (Ec) | 126 | - | - | - | - | - | - | - |
| C.V.(%) | - | 3.2529 | 1.8608 | 5.3973 | 2.1651 | 4.9101 | 10.3370 | 9.4415 |

ns, *, ** non-significant, significant at *P* < 0.05 and *P* < 0.01, respectively. PC= Percentage of Colonization, RWC= Relative Water Content, WSD= Water Saturate Deficit, ASC= Alcohol-Soluble Carbohydrate, WSC= Water-Soluble Carbohydrates.

**Supplementary Table S3 |** Continued

|  |  | Mean square | | | | | |
| --- | --- | --- | --- | --- | --- | --- | --- |
| S.O.V. | **df** | **MSI** | **Chlorophyll a** | **Chlorophyll b** | **Total chlorophyll (a + b)** | **Carotenoid** | **Grain Yield** |
| Repeat | 3 | ns | ns | ns | ns | ns | ns |
| Irrigation (I) | 2 | ** | ** | ** | ** | ** | ** |
| (Ea) | 6 | - | - | - | - | - | - |
| Cultivars (C) | 1 | ** | ** | ** | ** | ** | ** |
| (C ×I) | 2 | ns | * | * | ns | ns | ns |
| (Eb) | 9 | - | - | - | - | - | - |
| Biofertilizers (B) | 7 | ** | ** | ** | ** | ** | ** |
| (B × I) | 14 | * | * | ns | ** | * | ns |
| (B × C) | 7 | ns | ns | ns | ns | ns | ns |
| (B × I × C) | 14 | ns | ns | ns | ns | ns | ns |
| (Ec) | 126 | - | - | - | - | - | - |
| C.V.(%) | - | 2.4382 | 4.8353 | 12.8532 | 4.0104 | 6.6753 | 3.3425 |

ns, *, ** non-significant, significant at *P* < 0.05 and *P* < 0.01, respectively. MSI= Cell Membrane Stability Index.
